# Plants of Conservation Interest in a Protected Area: A Case Study of the Gran Sasso and Monti Della Laga National Park (Central Italy)

**DOI:** 10.3390/plants13121675

**Published:** 2024-06-17

**Authors:** Fabio Conti, Daniela Tinti, Fabrizio Bartolucci

**Affiliations:** 1 Centro Ricerche Floristiche dell’Appennino, Scuola di Bioscienze e Medicina Veterinaria, Università di Camerino—Parco Nazionale del Gran Sasso e Monti della Laga, Via Prov.le km 4,1, Barisciano, 67021 L’Aquila, Italy; fabio.conti@unicam.it; 2 Parco Nazionale del Gran Sasso e Monti della Laga, Ufficio Centro Ricerche Floristiche dell’Appennino, Via Del Convento 1, Assergi, 67010 L’Aquila, Italy; danielatinti@gransassolagapark.it

**Keywords:** conservation strategies, in situ/ex situ conservation, plant conservation, plant diversity, vascular flora

## Abstract

The National Park of Gran Sasso and Monti della Laga (PNGSL) is located in Central Italy and covers an area of 143.311 ha across three administrative regions (Abruzzo, Marche, and Lazio). It is the protected area hosting the highest number of vascular plants in both Europe and the Mediterranean basin. The plan of the park recognizes the need to establish a list of plants of conservation interest to prioritize for protection. The aim of this study is to identify plants (vascular and bryophytes) for inclusion on a protection list, taking into account their phytogeographic importance as well as the threat of extinction, and subsequently propose an original categorization (protection classes) suggesting specific conservation actions and measures. We used original criteria to select plants of conservation interest among the 2678 plant taxa listed in the national park. We identified 564 vascular plant species and subspecies (including nine hybrids) and one bryophyte to be included in the proposed protection list. The case study of the PNGSL could be a model for other protected areas.

## 1. Introduction

Conservation of biodiversity requires careful planning [1,2,3,4], as it is not possible to assist all species under threat, typically due to limited funding and human resources [5]. Priorities must therefore be established by making a list of plants of conservation interest at different levels [1,2,3]. It is critical to select conservation priorities and management strategies that are most effective and proper for the local area.

A consistent list of plants of conservation interest, even at a local level, can be deduced from red lists. The IUCN Categories and Criteria were developed to improve objectivity and transparency in assessing the conservation status of species, and thus to improve consistency and understanding among users [6]. In Italy, the more recently published red lists [7] include policy species (taxa listed in the annexes of the Habitats Directive 92/43/EEC and Bern Convention), taxa endemic to Italy [8,9], and a group of taxa of conservation concern (plants occurring in highly threatened habitats (e.g., wetlands and coastal habitats) and/or considered as EX, EW, or CR in the previous Italian red lists [10,11]. The conservation process is strictly linked to the distribution and biogeography of taxa [12]. It is important to simultaneously consider multiple criteria such as rarity, endemicity, and risk of extinction for the purposes of conservation prioritization [13]. Protected areas play a fundamental role in the conservation of plant diversity [14,15,16] by allowing space for the development of appropriate strategies for in situ [17,18] and ex situ protection [19,20], for the preservation of a single species or some selected policy species [21,22,23], and for drafting lists of plants of conservation interest [24,25,26,27]. The Gran Sasso and Monti della Laga National Park (hereafter, PNGSL) is the protected area with the highest number of vascular plants (species and subspecies) in both Europe and the Mediterranean basin [28].

The aim of the national park is to identify a list of plants of conservation interest to be included in the “Beni Ambientali Individui” [Individual Environmental Assets] (hereafter, BAI). The BAI, as defined by art. 16 of the Implementation legislation of the Plan, albeit with improper terminology, are emergencies of any type “recognized by national and international regulations, or identified by studies and research by the Park Authority or other competent subjects (institutional and otherwise)”.

The aim of this study is to select plants to be included in a protection list, as commissioned by the PNGSL. We considered not only the plants under threat included in the red lists or in the regional protection regulations but also plants worthy of note for their phytogeographical interest, which was determined by assessing their endemicity, rarity, disjunction, or range limit. Therefore, we proposed original criteria to select plants and five protection classes with different appropriate levels of knowledge, conservation measures, and actions for protection, management, and monitoring. We have also developed an analogous list of plants by adopting similar criteria and measures for the Abruzzo, Lazio, and Molise National Parks [29].

## 2. Results

Based on the objective criteria identified (Table 1), 564 vascular plant species and subspecies (including nine hybrids) and one bryophyte were included in the protection classes. For each species and subspecies, the taxonomic group, family, endemicity, protection class, criteria, inclusion in red lists, and relevant regional laws for the protection of flora or international conventions are reported (see File S1).

In total, 21% of the flora of the PNGSL are vascular plants included in the BAI. Among these, 165 taxa belong to the higher protection classes (A and B), and 12 can be considered probably extinct in the study area. This latter number is high mainly due to the destruction of the Campotosto peat bog in the first half of the 19th century, with the creation of the homonym artificial lake. Some of these plants, such as *Carex lasiocarpa* Ehrh., *C. elongata* L., *Comarum palustre* L., *Salix rosmarinifolia* L., etc., should be considered disappeared from the entire Apennines [9].

Some new populations of taxa included in the higher protection classes (A and B) are monitored every year. Furthermore, newly discovered taxa in the flora of the park, included in the higher protection classes, are usually monitored from the time of their discovery (e.g., *Huperzia selago* (L.) Bernh. ex Schrank and Mart., *Oxytropis ocrensis* and *Ranunculus lateriflorus* DC.).

**Figure 1:**
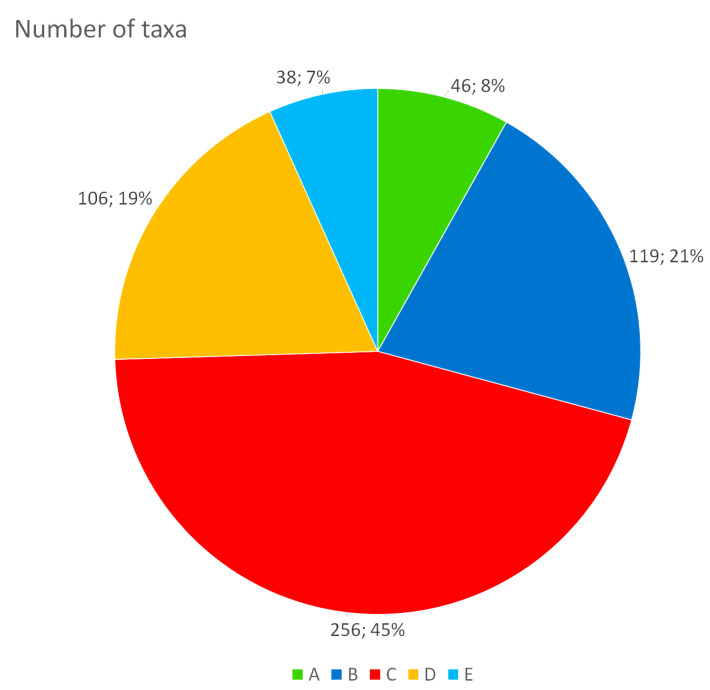
Number and percentage (%) of taxa included in each of the protection classes: A—highest conservation interest; B—high conservation interest; C—good conservation interest; D—highest to good conservation interest but with doubtful taxonomic value or belonging to critical groups of Italian flora; E—extinct or not confirmed to the study area.

**Figure 2:**
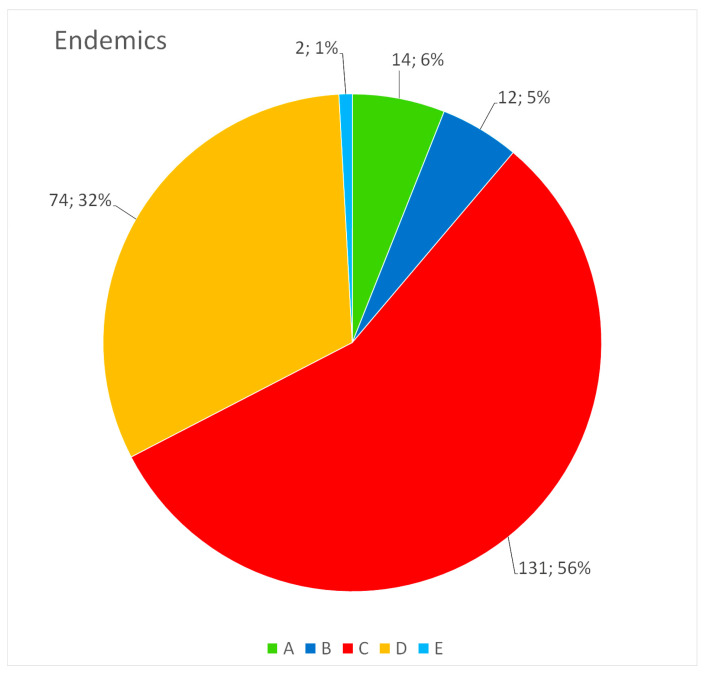
Number and percentage (%) of plants endemic to Italy included in each of the protection classes: A—highest conservation interest; B—high conservation interest; C—good conservation interest; D—highest to good conservation interest but with doubtful taxonomic value or belonging to critical groups of Italian flora; E—extinct or not confirmed to the study area.

The number of species and subspecies (including bryophytes) for each of the protection classes is as follows (Figure 1): A (46), B (119), C (256), D (106), E (38). Endemic plants are distributed throughout the different classes, as reported in Figure 2. Analyzing the 46 plants included in class A, based on preferential growth habitat, almost all of them live in humid environments (15) and mainly in peat bogs, little lakes, or pools (e.g., *Allium permixtum* Guss., *Carex canescens* L. subsp. *canescens, C. davalliana* Sm., *Elatine alsinastrum* L., *Juncus sp. pl., Ranunculus bariscianus* Dunkel, *R. giordanoi* F.Conti and Bartolucci, *R. lateriflorus* DC., *R. pedrottii* Spinosi ex Dunkel, *Salix pentandra* L. and *Vallisneria spiralis* L.). Other significant contingents are represented by high-altitude plants, which include species endemic to the Central Apennines such as *Adonis distorta* Ten., *Androsace mathildae* Levier, *Oxytropis ocrensis* F.Conti and Bartolucci, *Saxifraga italica* D.A.Webb and *Soldanella minima* Hoppe subsp. *samnitica* Cristof. and Pignatti. A third contingent is represented by steppe plants such as *Adonis vernalis* L., *Alyssum desertorum* Stapf, *Jacobaea vulgaris* Gaertn. subsp. *gotlandica* (Neuman) B.Nord., including some endemic taxa such as *Astragalus aquilanus* Anzal., *Gonliolimon tataricum* (L.) Boiss. subsp. *italicum* (Tammaro, Pignatti and Frizzi) Buzurović and *Stipa aquilana* Moraldo. The last group of plants is linked to nemoral environments in the past subjected to excessive forest cutting (*Astragalus penduliflorus* Lam., *Botrychium matricariifolium* (A.Braun ex Döll) W.D.J.Koch, *Buphthalmum salicifolium* L. subsp. *salicifolium*, *Orobanche salviae* F.W. Schultz, *Struthiopteris spicant* (L.) Weiss.).

Examining the plants belonging to the highest protection classes (A and B), it is evident that they live in the most threatened habitats, and therefore, their conservation involves the protection of the habitat in which they grow.

The populations of 32 species and subspecies included in the protection classes A, B, and E were monitored between 2012 and 2023. Some populations of *Allium permixtum* Guss., *Buphthalmum salicifolium* L. subsp. *salicifolium*, *Diphasiastrum complanatum* (L.) Holub, whose occurrences in the Park’s territory are due to the existence of old herbarium specimens housed in APP, CAME, and FI [30], were not found during field activities. For all the monitored populations, data concerning the species’ localization, population size, and threats and pressures were collected. These data are collected in a specific database that the technicians of the park have at their disposal and can consult when an intervention is proposed that could damage species or habitats within the park’s territory. Upon specific request, and subject to authorization from the Park Authority, even private persons involved in activities within the Park’s territory can request to view this data. Some populations of species particularly at risk due to ongoing pressures have been monitored several times over the years, confirming their good state of conservation thanks also to the specific measures implemented (i.e., *Orobanche salviae* F.W.Schultz). The information collected during monitoring relates to rare or at-risk species and is therefore particularly sensitive, which is why we do not make it available in the following table (Table 2).

## 3. Discussion

This study, at first funded by PSR Abruzzo Region (2007–2013—Axis 3—Call for Measure 3.2.3), was carried out as part of the drafting of the management plans of Natura 2000 sites (natural sites in Europe protected under the EU Birds and Habitats Directives) and subsequently updated on behalf of the PNGSL. The Decree of Italian Republic President (DPR 357/07) establishes that, in the case of Natura 2000 sites falling within Protected *Areas sensu* L. 394/91, the plan of the site, with the appropriate conservation measures pursuant to the Habitat Directive, must be integrated into the Management Plan. Furthermore, the latter must respect the Regulation already drawn up for the Protected Area itself, respectively, by Arts. 11 and 12 of the framework law on protected areas. The Plan of the PNGSL, adopted and approved by the Governing Council Resolution of the National Park n. 35/99 of 21 December 1999 and by the Abruzzo, Marche, and Lazio administrative regions, was published in the “Gazzetta Ufficiale” Part II n. 124 of 22/10/2020. The protection of plant species occurring in the area is among the aims of the Park, in particular, those that are rare and at risk of extinction. The identification of these noteworthy floristic taxa (included in the BAI) and the related habitat, as well as the implementation of appropriate forms of protection, also find application in the Park Plan. Regarding flora, the Plan provides that “all endemic, relict, rare or endangered species included in the National and Regional Red Lists, as well as species of Community Importance (identified by the Habitat Directive) and of International Conventions” should be protected. The need for conservation of these plants is highlighted by the Plan, which “recognizes the need to subject them to maximum protection, even if located in areas that do not coincide with the Reserves”. In fact, the zoning of the Park for the Management Plan was obtained according to more general criteria, which did not consider the specific presence of species of conservation interest [31]. The integration between zoning and art. 16 for the BAI guarantees the optimal protection of species of conservation interest. The Park Regulations also “specify, integrate and, if appropriate, enrich the above list and regulate in detail the methods of protection”, based on the progress of knowledge of the subject.

Any economic activities within the Park, promoted mostly by private individuals, require an evaluation often based on the presence of BAIs. The correct application of the management indications for the plants included in the protection classes as indicated in Table 3, and provided by the Offices of PNGSL, allowed the implementation of an exhaustive and standardized assessment of the impacts derived from activities, works, and events and in general for any type of authorization request received by the Park.

For all the monitored populations included in the BAIs, fundamental data were collected to understand, maintain and, where possible, improve the conservation status of the plant species involved. These data, entered into a specific database, are available to Park technicians. A recent case is worth mentioning here. In 2020, the Park Authority received a request for authorization from a company that deals with the distribution of electricity across the national territory for the reconstruction of an overhead power line section, involving the replacement of support elements. The Park Authority Office, responsible for issuing the authorization, consulted, as is now internal practice, the distribution of the BAIs, noting the presence of some populations of *Astragalus aquilanus* close to the intervention site. The Office requested an in-depth check before starting the work, which revealed the presence of a not known population of *Astragalus aquilanus* (All. II* of Habitat Directive, included in A class of BAI) exactly in the intervention site. Consequently, suitable measures and modifications to the project have been defined in order to safeguard the population.

The need to propose a list of plants to be protected, such as the BAIs, is closely linked to the fact that there are no European, national, or even local regulations that can be considered satisfactory for the protection of endemic or rare flora of conservation and biogeographical interest.

At the local level, the adoption of a list of species of conservation interest (BAIs) seems to be a good solution, especially for protected areas, which often do not have useful tools for the protection of flora, apart from the few plants included in the Habitats Directive. In Italy, only 115 taxa (104 vascular plants, 10 bryophytes, 1 lichen) are included in Annexes II, IV, and V of the Habitats Directive [32]: a very small number compared to the high floristic richness of Italy, in which 8241 species and subspecies are registered, including 1702 taxa endemic to Italy or narrowly endemic to restricted areas [9]. At the regional level, the use of the list of Abruzzo administrative region is quite inappropriate because the only law is old and does not respond to the current needs that knowledge requires (regional laws n. 45/1979 and 66/1980). We proposed a regional law with a list of vascular plants to protect [33], recently updated but not yet implemented by the regional authorities. At the national level, there is no regulation on this concern. For this reason, extremely rare and threatened species are not adequately protected (i.e., *Goniolimon tataricum* subsp. *italicum*, strictly endemic to the L’Aquila basin, which is not protected by any Law or Directive and live outside any protected area). There is a felt need to promote both regional and national lists of plants to be protected which can be updated annually or every two years. It can be conducted through a review, the nomenclature, distribution and belonging classes of the species considered, following the French example (Liste des espèces végétales protégées sur l’ensemble du territoire français métropolitain in Inventaire National du Patrimoin Naturel, 2023) or the Spanish one by the Law 42/2007, of Natural Heritage and Biodiversity.

## 4. Conclusions

The conservation of plant biodiversity is a central theme and a crucial challenge globally. The main causes of the loss of biodiversity are attributable to the growing consumption of land with consequent reduction and fragmentation of natural habitats, climate change, and, no less importantly, the often devastating impact that invasive alien species have on native species and natural ecosystems. To combat the loss of plant biodiversity, correct, systematic, and conscious planning of conservation strategies is necessary, accompanied by concrete in situ and ex situ protection actions. Unfortunately, international, national, or local laws concerning the protection of native flora in Italy underestimate the plant biodiversity of our territory, making it impossible to concretely safeguard and conserve the rarest and most endangered species. From this perspective, the creation of a list of plants of conservation interest, developed, managed, and updated by a National Park Authority, becomes an essential tool for the knowledge, management, and protection of plant biodiversity at a local level.

## 5. Materials and Methods

Thanks to the institution of the Apennine Floristic Research Center in 2001, managed by the University of Camerino (UNICAM) and the PNGSL [34], the vascular flora of the Park has been critically studied and continuously updated [35]. Recently, 14 new taxa (species and subspecies) have been recorded for the protected area: *Carex strigosa* Huds., *Ranunculus pedrottii* Spinosi ex Dunkel [36], *Anthyllis apennina* F.Conti and Bartolucci, *Taraxacum pudilii* Štěpánek and Kirschner [37], *Oxytropis ocrensis* F.Conti and Bartolucci, *Aubrieta deltoidea* (L.) DC., *Opuntia scheeri* F.A.C.Weber, *Trachelium caeruleum* L. subsp. *caeruleum* [38], *Anacamptis berica* Doro [39], *Astragalus austriacus* Jacq., *Festuca bosniaca* Kumm. and Sendtn. subsp. *bosniaca*, *Ranunculus lateriflorus* DC., *Soldanella minima* subsp. *samnitica* Cristof. and Pignatti, *Erigeron annuus* subsp. *strigosus* (Muhl. ex Willd.) Wagenitz [40,41]. Furthermore, *Pulmonaria officinalis* L. subsp. *officinalis* reported by [42] was not cited by [35] in the recent update of the Park’s flora, while *Cytinus hypocistis* (L.) L. subsp. *hypocistis* and *Carex microcarpa* Bertol. ex Moris should be excluded from the Park [43]. Currently, 2678 taxa (species and subspecies) are known, 233 of which are endemic to Italy. Among these, 114 are endemic to the Central Apennines and 12 are narrow endemics to the PNGSL. All the floristic data (from bibliographic and herbarium sources) concerning vascular plants and bryophytes of the Park were entered into a geodatabase using File Maker Pro software version 19.2.2.234. The geodatabase, continuously updated, includes data for the Abruzzo administrative region and the neighboring areas in Molise, Lazio, and Marche included within PNGSL and Abruzzo, Lazio, and Molise National Park. The locations, coming from herbarium samples or bibliography, of the species reported in the Abruzzo geodatabase, are georeferenced [44].

Starting from the checklist of the Park’s flora, we identify the plants to be included in the protection list based on the following criteria:

- Endemic species to Italy [8,9];
- Exclusive species of one of the administrative regions in which the Park territory falls (species also distributed outside the national borders, but in Italy present in only one administrative region [9]);
- Exclusive species to the Park (species distributed also outside the national borders, but in Italy present only in the Park [28,35]);
- Very rare or rare plants according to the current level of knowledge relating to Central Italy;
- Plants with disjointed populations (species present in the Park with a portion detached from the main distribution range);
- Plants listed in regional laws concerning the protection of the flora (L. R. Abruzzo n. 45 of 11/09/1979, L. R. Abruzzo n. 66 of 20/06/1980; L. R. Marche n. 8 of 10.01.1987; L. R. Lazio n. 61 of 19.09.1974);
- Plants listed in the international regulations (Habitat Directive 92/43 EEC; Convention on the Conservation of Wild Life and Natural Habitats, Bern 1979; Convention on International Trade in Endangered Species of Fauna and Flora CITES, Washington 1973);
- Plants included in the Italian Red List [45,46].

To direct actions and measures more specifically, 5 classes of protection and related criteria were proposed, labeled as A, B, C, D, and E (Table 1). For each class, the desirable level of knowledge, the proposed conservation measures, the actions for their protection and management, and the appropriate monitoring activities were indicated (Table 3). The occurrence of even just one of the established criteria determines a plant’s belonging to the relevant class.

For the most threatened species or those included in classes A or B, we planned and carried out, from 2012 to 2023, field activities with the aim of tracing populations mentioned in the bibliography and monitoring their state of conservation.

During the monitoring of each species’ population, information about the localization, surface area, population size, habitat, main pressures/threats, and conservation measures have been collected. The populations monitored were analyzed according to the specific protocols developed in accordance with what was proposed by the SBI (Italian Botanical Society) and ISPRA (Istituto Superiore per la Protezione e la Ricerca Ambientale) during the third report under art. 17 of the Habitats Directive [32].

The nomenclature of the plants included in the protection list follows the checklist of native Italian vascular flora [9] and the Italian checklist of bryophytes [47].

## Figures and Tables

**Table 1:** Criteria for defining the conservation classes of plants of conservation interest. Classes: A—highest conservation interest; B—high conservation interest; C—good conservation interest; D—highest to good conservation interest but with doubtful taxonomic value or belonging to critical groups of Italian flora; E—extinct or not confirmed to the study area.

Class	Codes	Criteria
A	a1	Endemic to the PNGSL and immediately adjacent territories.
	a2	In Italy, exclusive to PNGSL and immediately adjacent territories.
	a3	Endemic to the Central Apennines, provided they are rare and/or subjected to real threats.
	a4	In Italy, exclusive to one of the administrative regions affected by the PNGSL (Abruzzo, Lazio, and Marche), provided they are rare or subject to real threats.
	a5	Protected by international regulations, provided they are rare or subject to real threats.
	a6	Included in the IUCN Global Red List and/or in the Italian red list in the CR category.
	a7	Extremely rare or in the process of rarefaction according to the current level of knowledge relating to Central Italy.
B	b1	Endemic to one of the administrative regions affected by the PNGSL (Abruzzo, Lazio, and Marche), provided they are not common.
	b2	In Italy, exclusive to one of the administrative regions affected by the PNGSL (Abruzzo, Lazio, and Marche), provided they are not common.
	b3	Rare and of particular phytogeographic value as they are endemic to the Central Apennines or present in the Park and neighboring territories with disjointed populations, relics, or at the limit of the range.
	b4	Included in the IUCN Global Red List and/or in the Italian red list in the EN category.
	b5	Protected at the regional level in at least one of the administrative regions affected by the Park’s territory (Abruzzo—LR 45 of 11.09.1979 and 66 of 20.06.1980; Marche—LR 8 of 10.01.1987; Lazio—LR 61 of 19.09.1974), provided they are rare or subject to real threats.
	b6	Elsewhere they are widespread, but present in the Park in limited numbers (≤4).
C	c1	Italian endemic species with a wide range.
	c2	Included in the IUCN Global red list and/or in Italian red list in the VU category (vulnerable).
	c3	Protected by international regulations, provided they are common and not subjected to real threats.
	c4	Protected at the regional level (Abruzzo—LR 45 of 11.09.1979 and 66 of 20.06.1980; Marche—LR 8 of 10.01.1987; Lazio—LR 61 of 19.09.1974), provided they are common and not subjected to real threats (excluding very common entities).
D	d1	Plants that have at least one of the requisites to be ascribed to the protection classes A, B, C, or E, but of doubtful taxonomic value or belonging to critical groups of Italian flora.
	d2	Plants that have at least one of the requisites to be ascribed to protection classes A, B, C, or E, but whose indication in the Park’s territory is doubtful.
E	e1	Extinct in the study area, whose historical presence is supported by herbarium samples.
	e2	Extinct in the study area, whose historical presence is supported by the reliable literature data.
	e3	Not recently confirmed.

**Table 2:** Taxa monitored between 2012 and 2023.

Family	Italian Endemic Taxa	Taxon	Monitored (Year)	Protection Class
Ranunculaceae	E	*Adonis distorta* Ten.	2012, 2013	A
Ranunculaceae		*Adonis vernalis* L.	2012, 2014, 2021	A
Amaryllidaceae		*Allium permixtum* Guss.	2012, 2014, 2021, 2022	A
Primulaceae	E	*Androsace mathildae* Levier	2012, 2016	A
Asteraceae		*Artemisia eriantha* Ten.	2012, 2013	B
Fabaceae	E	*Astragalus aquilanus* Anzal.	2013, 2014, 2020	A
Asteraceae		*Buphthalmum salicifolium* L. subsp. *salicifolium*	2014	A
Buxbaumiaceae		*Buxbaumia viridis* (Lam. and DC.) Moug. and Nestl.	2014	A
Brassicaceae	E	*Cardamine apennina* Lihová and Marhold	2012	B
Cyperaceae		*Carex canescens* L.	2021	A
Cyperaceae		*Carex firma* Host	2013	A
Colchicaceae		*Colchicum bulbocodium* Ker Gawl. subsp. *versicolor* (Ker Gawl.) K.Perss.	2014	B
Brassicaceae		*Conringia austriaca* (Jacq.) Sweet	2019	B
Lycopodiaceae		*Diphasiastrum complanatum* (L.) Holub	2014, 2021, 2022, 2023	E
Brassicaceae		*Draba dubia* Suter subsp. *dubia*	2014	A
Elatinaceae		*Elatine alsinastrum* L.	2014	A
Fabaceae	E	*Genista pulchella* Vis. subsp. *aquilana* F.Conti and Manzi	2012	A
Plumbaginaceae	E	*Goniolimon tataricum* (L.) Boiss. subsp. *italicum* (Tammaro, Pignatti and Frizzi) Buzurović	2012	A
Lycopodiaceae		*Huperzia selago* (L.) Bernh. ex Schrank and Mart. subsp. *selago*	2021, 2023	B
Asteraceae		*Jacobaea vulgaris* Gaertn. subsp. *gotlandica* (Neuman) B.Nord.	2013, 2020	A
Fabaceae	E	*Lathyrus apenninus* F.Conti	2012, 2013, 2014	B
Fabaceae		*Ononis rotundifolia* L.	2012	A
Orobanchaceae		*Orobanche salviae* F.W.Schultz	2013, 2018	A
Fabaceae	E	*Oxytropis ocrensis* F.Conti and Bartolucci	2022	A
Lentibulariaceae	E	*Pinguicula vulgaris* L. subsp. *vestina* F.Conti and Peruzzi	2012	A
Rosaceae		*Potentilla nitida* L.	2013	A
Ranunculaceae		*Pulsatilla montana* (Hoppe) Rchb. subsp. *montana*	2012, 2013	B
Ranunculaceae		*Ranunculus lateriflorus* DC.	2022	A
Lamiaceae		*Salvia verticillata* L. subsp. *verticillata*	2022	B
Saxifragaceae	E	*Saxifraga italica* D.A.Webb	2013	A
Brassicaceae		*Sinapis pubescens* L. subsp. *pubescens*	2019	B
Orchidaceae		*Traunsteinera globosa* (L.) Rchb.	2012, 2022	A
Ericaceae		*Vaccinium uliginosum* L. subsp. *microphyllum* (Lange) Tolm.	2012, 2013, 2023	A

**Table 3:** Management indications for the plants included in the protection classes.

Class A	Desirable level of knowledge	Detailed knowledge of the populations (location, extent, and number of individuals) of each location within the Park. Knowledge on the biology and ecology of the species. Detailed assessment of any real and potential risks, natural and/or of anthropogenic origin, which threaten the specific population and the genetic exchange between populations. Preparation of protocols for in situ and ex situ conservation.
	Proposed conservation measures	In situ (interventions for the elimination of or reduction in pressures, possible restocking, actions for the prevention of threats). Ex situ (conservation of germplasm; reproduction and cultivation in authorized structures). Training, information, teaching, awareness raising aimed at different target audiences (including administrations).
	Actions for protection and management	Interventions or activities of any kind that exercise or that could have an impact, even indirect or presumed, on the species or sites in which they are present, are not authorized in any way. Interventions or activities that affect habitats potentially suitable for hosting the species must be evaluated through field surveys carried out by expert botanists in the season favorable to the observation of the species. This allows experts to exclude with absolute certainty the presence of the entities on site or the possibility that entities can colonize the site in a relatively short time, coming from populations located at a short distance. This is especially the case if these populations are themselves threatened and/or fragmented. Requests for authorization for the collection of plants or parts of plants can be evaluated only if aimed at the conservation of the species, giving priority to what is provided in the two previous columns.
	Monitoring	Every 3 (annual species) to 5 years (population size, trend, pressures and threats) and research in the field for new localities.
Class B	Desirable level of knowledge	Detailed knowledge of some significant populations (location, extent and number of individuals) within the Park. Knowledge on the biology and ecology of the species. Detailed assessment of any real and potential risks, of natural and/or anthropogenic origin, which threaten the specific population and the genetic exchange between populations. Preparation of protocols for in situ and ex situ conservation.
	Proposed conservation measures	In situ (interventions for the elimination of or reduction in pressures, actions for prevention of threats). Ex situ (conservation of germplasm; reproduction and cultivation in authorized structures). Training, information, teaching, awareness raising aimed at different target audiences (including administrations).
	Actions for protection and management	Interventions or activities of any kind that exercise or that could have an impact, even indirect or presumed, on the species or sites in which they are present, are not authorized in any way. Requests for authorization to collect plants or parts of plants can only be evaluated if they are aimed at the conservation of the species or for scientific purposes.
	Monitoring	Every 3 (annual species) to 5 years (population size, trend, pressures and threats) and research in the field for new localities.
Class C	Desirable level of knowledge	Knowledge of the species distribution within the Park and possible threatening factors. Knowledge on the biology and ecology of the species.
	Proposed conservation measures	Training, information, teaching, awareness raising aimed at different target audiences (including administrations).
	Actions for protection and management	To be determined in relation to the presence and abundance of the species in question, together with the overall assessment of the natural state of the site.
	Monitoring	
Class D	Desirable level of knowledge	Studies aimed at resolving doubt about the presence of the species in the Park or its taxonomic issues for the re-attribution to the right class from A to E.
	Proposed conservation measures	
	Actions for protection and management	
	Monitoring	
Class E	Desirable level of knowledge	Evaluation of the reliability of the reports and field verification.
	Proposed conservation measures	The necessary conservation measures will be evaluated in case of re-discovery of the species and of the relative protection class to which it can be assigned. Training, information, teaching, awareness raising aimed at different target audiences (including administrations).
	Actions for protection and management	Interventions or activities that affect the sites where the species had been indicated in the past must be carefully evaluated by means of field surveys carried out by expert botanists in the season favorable to the observation of the species in order to exclude with absolute certainty the presence of the species in the site.
	Monitoring	Every 3 (annual species) to 5 years; field activities must be planned with the aim of verifying the localities of ancient reporting of the species.

## Data Availability

The data presented in the current study are available within the article.

## Supplementary Materials

The following supporting information can be downloaded at: https://www.mdpi.com/article/10.3390/plants13121675/s1, File S1: list of taxa of conservation interest in alphabetical order.
